# Occurrence and management of thrombosis recurrence and bleeding in low-molecular-weight heparin-treated patients with cancer-associated thrombosis: a French nationwide cohort study

**DOI:** 10.1016/j.rpth.2024.102642

**Published:** 2024-11-26

**Authors:** Isabelle Mahé, Gaelle Gusto, Nadia Quignot, Artak Khachatryan, Jose Chaves, Audrey Moniot, Lucas Andre, Sylvain Van Roy, Ruth Mokgokong, Laurent Bertoletti

**Affiliations:** 1 Université Paris Cité, Paris, France; 2 Assistance Publique des Hôpitaux de Paris, Hôpital Louis Mourier, Service de Médecine Interne, Inserm Unité Mixte de Recherche_S1140, Innovations Thérapeutiques en Hémostase, Paris, France; 3 Certara France, Paris, France; 4 Certara UK, London, United Kingdom; 5 Pfizer SLU, Madrid, Spain; 6 Pfizer SAS, Paris, France; 7 Pfizer Ltd, Tadworth, United Kingdom; 8 Service de Médecine Vasculaire et Thérapeutique, Centre Hospitalo Universitaire de St-Etienne, Saint-Etienne, France; 9 Institut national de la santé et de la recherche médicale, Unité Mixte de Recherche1059, Equipe Dysfonction Vasculaire et Hémostase, Université Jean-Monnet, Saint-Etienne, France; 10 Institut national de la santé et de la recherche médicale, Centre d'Investigation Clinique-1408, Centre Hospitalo Universitaire de Saint-Etienne, Saint-Etienne, France

**Keywords:** cohort studies, hemorrhage, heparin, low-molecular-weight, neoplasms, recurrence, venous thromboembolism

## Abstract

**Background:**

Rates of venous thromboembolism (VTE) recurrence and bleeding remain high in patients with cancer who are prescribed anticoagulants (ACs) such as low-molecular-weight heparin (LMWH) after an initial VTE event.

**Objectives:**

To identify patient characteristics associated with VTE recurrence and bleeding in patients receiving LMWH for cancer-associated VTE and to explore secondary AC management and clinical outcomes in these patients.

**Methods:**

An observational study was conducted using nationwide French data for adults with active cancer who were hospitalized with VTE in 2013-2018 and were reimbursed for LMWH ≤ 30 days after hospital discharge. The main outcomes were VTE recurrence and bleeding. For both outcomes, the proportions of patients who experienced the outcome were calculated for different patient characteristics. AC switching following VTE recurrence and bleeding was tracked using Anatomical Therapeutic Chemical codes.

**Results:**

A total of 31,771 patients received LMWH, of whom 1925 (6.1%) experienced VTE recurrence and 1804 (5.7%) bleeding. Most recurrent VTE and bleeding events occurred within 6 months after the initial VTE event. The proportion of patients with VTE recurrence and bleeding varied between cancer types. Most patients who experienced VTE recurrence or bleeding continued to receive LMWH. Eleven percent of patients with VTE recurrence experienced a further recurrent VTE event within 3 months.

**Conclusion:**

More than 10% of patients who received LMWH for cancer-associated VTE experienced VTE recurrence or bleeding. AC management options in this patient population should be prospectively assessed in clinical trials.

## Essentials

• Anticoagulant (AC) treatment is challenging in cancer patients with venous thromboembolism (VTE).
• We studied clinical outcomes and AC management in this patient group using nationwide French data.
• 6.1% of patients receiving low-molecular-weight heparin had VTE recurrence, and 5.7% had bleeding.
• Most patients with VTE recurrence continued to receive low-molecular-weight heparin.

## 1 Introduction

Venous thromboembolism (VTE) is common in patients who have cancer or who have undergone cancer surgery [[1], [2], [3]]. Importantly, VTE is associated with an increased risk of death in patients with cancer. In a study based on the population-based Scandinavian Thrombosis and Cancer cohort, cancer-associated VTE increased the risk of death more than 3-fold compared with the presence of cancer alone [4]. Recurrence of VTE in patients with cancer further increases the risk of death [5].

For the past 20 years, anticoagulant (AC) treatment with low-molecular-weight heparin (LMWH) has been the standard of care for VTE in patients with cancer [6]. However, treatment guidelines now recommend direct oral ACs (DOACs) as an alternative to LMWH [[7], [8], [9], [10], [11], [12], [13]]. Though it can prevent VTE from recurring, AC treatment can cause serious or even fatal bleeding. Patients with cancer-associated VTE have increased risks of both bleeding and VTE recurrence due to the complex interplay between cancer-, treatment-, and patient-related factors [[14], [15], [16], [17]]. In a prospective follow-up study, the 12-month incidence of VTE recurrence in patients receiving heparin with warfarin bridging after an initial VTE event was 21% in patients with cancer and 7% in patients without cancer [14]. In the same study, the 12-month incidence of major bleeding was also higher in patients with cancer (12%) than in those without cancer (5%) [14].

AC treatment of cancer-associated VTE must carefully balance the risk of VTE recurrence against the risk of bleeding [8]. Available data suggest that rates of VTE recurrence typically exceed those of bleeding. In a meta-analysis of patients receiving AC treatment for cancer-associated VTE, the rate of VTE recurrence was 24 per 100 patient-years, and the rate of major bleeding was 13 per 100 patient-years [18]. The case fatality rate was also higher for recurrent VTE than for major bleeding (15% vs 9%) but did not differ significantly between different AC regimens. A second meta-analysis of DOAC trial data similarly found that the rate of recurrent VTE at 6 months in patients with cancer-associated VTE was higher than the corresponding rate of major bleeding for both DOACs (5.6% vs 4.8%) and LMWH (8.3% vs 3.5%) [19].

Because ACs can increase the risk of bleeding, patients receiving them need frequent monitoring [20]. However, optimal management of patients with cancer who experience bleeding or VTE recurrence during AC treatment is unclear, and guidance is limited. Some smaller studies have supported increasing the AC dose in patients who experience VTE recurrence [21,22], and American Society of Hematology and French guidelines both recommend that physicians increase the LMWH dose to a supratherapeutic level in these patients [8,12,23]. However, for patients who have bleeding events while on LMWH or other ACs, there are no clear guidelines for physicians to adjust the AC dose or to switch patients to another AC.

Despite treatment advances and updates to treatment guidelines, rates of VTE recurrence and bleeding remain high in patients with cancer who are prescribed ACs after an initial VTE event [24,25]. To better understand the factors contributing to these risks, we used a nationwide health database to determine whether cancer type and other clinical and demographic characteristics were associated with rates of VTE recurrence and bleeding in patients with cancer who were prescribed LMWH after an initial VTE event. We also explored secondary AC management and clinical outcomes in patients who experienced VTE recurrence or bleeding. We focused on patients prescribed LMWH because it was the recommended AC during the study period.

## 2 Methods

### 2.1 Study design, data source, and study population

**Figure 1:**
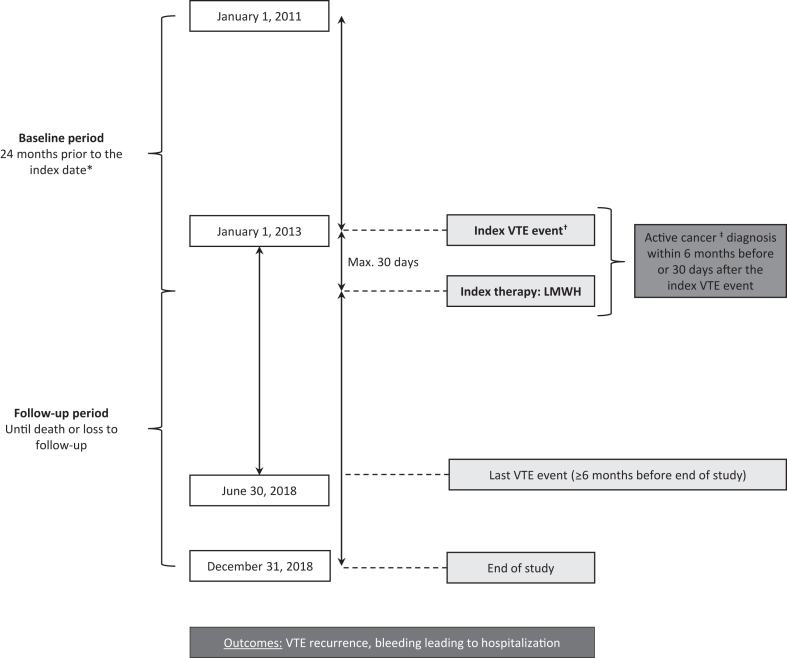
Overall study design. ∗Date of the first low-molecular-weight heparin (LMWH) dispensation record within 30 days after the index venous thromboembolism (VTE) event. ^†^First ICD-10 diagnosis of deep vein thrombosis or pulmonary embolism as (1) a primary hospital discharge diagnosis or (2) a secondary diagnosis associated with a concomitant procedure. ^‡^Cancer diagnosis or cancer treatment (chemotherapy, hormonotherapy, immunotherapy, radiation, or cancer-related surgery).

The study design and data source were reported previously [26]. Briefly, this was an observational study (EU PAS registration number: EUPAS35888) conducted using data from the French national health data system, *Système National des Données de Santé* (SNDS), which covers approximately 99% of the French population. The study population comprised adults (≥18 years) with active cancer who were hospitalized with VTE as a primary discharge diagnosis or secondary diagnosis associated with a concomitant procedure between January 1, 2013, and June 30, 2018, and who were reimbursed for LMWH within 30 days after hospital discharge [25,26]. The study period was selected to precede the widespread adoption of DOACs, which were first included in French treatment guidelines in 2021 [27]. The first VTE event during the study period was designated the index VTE event and the date of the first LMWH reimbursement within 30 days after the index VTE event was designated the index date (Figure 1). Active cancer was defined as a cancer diagnosis or cancer treatment (chemotherapy, hormonotherapy, immunotherapy, radiation, or cancer-related surgery) within 6 months before or 30 days after the index VTE event [26]. International Classification of Diseases 10th revision (ICD-10) codes (I26.0xxx, I26.9xxx, I80.1xxx, I80.2xxx, I80.3xxx, I80.9xxx, I82.1xxx, I82.2xxx, I82.8xxx, and I82.9xxx) and *Classification Commune des Actes Médicaux* diagnostic procedure codes (DFQH001, DHQH001 DHQH002, DHQH003, DHQH004, DHQH005, DHQH007, DHQM002, ECQH010, ECQH011, EFQM001, EJQM001, EJQM003, EJQM004, EKQH001, EMQH001, and ZBQH001) were used to identify patients with a VTE (deep vein thrombosis or pulmonary embolism) diagnosis [26]. The use of LMWH was identified using Anatomical Therapeutic Chemical (ATC) classification system codes. Exclusion criteria were reported previously [26].

### 2.2 Study outcomes

The main study outcomes were VTE recurrence (hospitalization with deep vein thrombosis or pulmonary embolism as a primary ICD-10 diagnosis >7 days after the index date) and bleeding (hospitalization with bleeding as a primary ICD-10 diagnosis anytime after the index date; ICD-10 codes used to identify cases of bleeding are listed elsewhere [26]). Follow-up started on the index date and continued until the earliest of the following: death (in or outside the hospital); loss to follow-up; treatment switching, discontinuation, or interruption; and the end of the follow-up period (December 31, 2018).

For both VTE recurrence and bleeding, clinical and demographic characteristics were compared between patients who experienced an event and those who did not experience an event. For each clinical and demographic characteristic, percentages of patients who experienced VTE recurrence or bleeding were also calculated.

Type of cancer and presence of metastatic disease at baseline were evaluated using all ICD-10 codes for cancer identified in the 6 months before and 30 days after the index date. Cancer types were categorized into different levels of VTE risk based on the Khorana score [28] and were nonexclusive (an individual patient could be included under more than 1 cancer type). Patients who developed metastatic disease more than 30 days after the index date were excluded from the analysis of metastatic disease status.

Baseline comorbidities were identified using ICD-10 codes extracted from hospital diagnoses (recorded during the 24 months before the index date) and from SNDS data on long-term disease (collected in the 5 years before the index date). The age-adjusted Charlson Comorbidity Index, which is used to predict 1-year mortality for patients with various comorbid conditions [29], was calculated. History of bleeding was defined as a bleeding event in the 24 months or 3 months (recent history) before the index date. Concomitant antiplatelet use in the 90 days before or after the index date was identified using ATC codes.

Secondary AC management after a recurrent VTE or bleeding event was examined with a focus on changes in the ACs patients received within the first month after the recurrent VTE or bleeding event. ACs were identified using ATC codes. Follow-up for clinical outcomes after AC switching was from the date of the first recurrent VTE event until death, loss to follow-up, or the end of the follow-up period (whichever was first).

### 2.3 Statistical analysis

Descriptive statistics were calculated for patient demographics, clinical characteristics, and study outcomes. Results are presented as mean (SD) or median (IQR) for continuous variables and *n* (%) for categorical variables. Data for different subgroups of patients were compared by chi-squared test (categorical variables) or analysis of variance (ANOVA; continuous variables). Treatment patterns and clinical outcomes after a first recurrent VTE event were plotted as a Sankey diagram [30]. To comply with *Commission Nationale de l'Informatique et des Libertés* data privacy requirements and protect patient confidentiality, precise numbers and percentages are not reported where the number of patients was <10.

## 3 Results

### 3.1 Patient selection and characteristics

During the study period, 39,023 patients with active cancer had a VTE event. Of these patients, 31,771 (81.4%) were prescribed LMWH as their initial AC [25] and were included in the present analysis. Smaller numbers of patients were prescribed a DOAC – apixaban (*n* = 678) or rivaroxaban (*n* = 2259) – as their initial AC [25]. The remaining patients were prescribed unfractionated heparin/fondaparinux (*n* = 2724) or a vitamin K antagonist (VKA; *n* = 1591). The most common cancer types in the patient sample were lung cancer (*n* = 7899; 24.9%), colorectal cancer (*n* = 5358; 16.9%), and breast cancer (*n* = 4381; 13.8%; Table 1).

**Table 1:** Characteristics of patients with and without recurrent venous thromboembolism.

Characteristic	LMWH overall (*N* = 31,771)	VTE recurrence during follow-up								
		Anytime during follow-up (*n* = 1925, 6.1%)	0-1 mo (*n* = 586, 30.4%)	>1-3 mo (*n* = 399, 20.7%)	>3-6 mo (*n* = 271, 14.1%)	>6-12 mo (*n* = 290, 15.1%)	>12-18 mo (*n* = 141, 7.3%)	>18 mo (*n* = 238, 12.4%)	No VTE recurrence (*n* = 29,846, 93.9%)	*P* value^a^
Age at index date (y)										
Mean (SD)	66.3 (13.2)	64.2 (13.2)	65.0 (13.6)	62.6 (13.5)	64.2 (13.2)	65.3 (13.1)	61.7 (11.5)	65.4 (12.2)	66.4 (13.2)	<.0001
Median (IQR)	67.0 (58.4-75.9)	64.9 (55.8-73.5)	65.1 (56.5-74.5)	63.2 (53.3-71.7)	64.3 (55.5-74.0)	65.9 (57.2-74.7)	63.7 (53.7-69.8)	66.3 (58.0-73.7)	67.2 (58.5-76.0)	
Male, *n* (%)	16,189 (51.0)	982 (51.0/6.1)	301 (21.4/1.9)	216 (54.1/1.3)	131 (48.3/0.8)	150 (51.7/0.9)	65 (46.1/0.4)	119 (50.0/0.7)	15,207 (51.0/93.9)	.79
Deprivation index, *n* (%)^b^										
Q4	11,385 (35.8)	659 (34.2/5.8)	191 (32.6/1.7)	132 (33.1/1.2)	105 (38.8/0.9)	99 (34.1/0.9)	48 (34.0/0.4)	84 (35.3/0.7)	10,726 (35.9/94.2)	<.0001
Q5	1657 (5.2)	94 (4.9/5.7)	22 (3.8/1.3)	20 (5.0/1.2)	15 (5.5/0.9)	16 (5.5/1.0)	7 (5.0/0.4)	14 (5.9/0.8)	1563 (5.2/94.3)	
Type of VTE, *n* (%)										
DVT alone	13,109 (41.3)	764 (39.7/5.8)	244 (41.6/1.9)	167 (41.9/1.3)	123 (45.4/0.9)	97 (33.5 0.7)	54 (38.3/0.4)	79 (33.2/0.6)	12,345 (41.4/94.2)	.004
PE (with or without DVT)	18,662 (58.7)	1161 (60.3/6.2)	342 (58.4/1.8)	232 (58.2/1.2)	148 (54.6/0.8)	193 (66.6/1.0)	87 (61.7/0.5)	159 (66.8/0.9)	17,501 (58.6/93.8)	
Rate of incident VTE by cancer type, *n* (%)										
Very high-risk										
Brain	1149 (3.6)	77 (4.0/6.7)	21 (3.6/1.8)	20 (5.0/1.7)	6 (2.2/0.5)	14 (4.8/1.2)	10 (7.1/0.9)	<10	1072 (3.6/93.3)	.13
Pancreatic	2541 (8.0)	148 (7.7/5.8)	58 (9.9/2.3)	40 (10.0/1.6)	19 (7.0/0.7)	14 (4.8/0.6)	<10	10 (4.2/0.4)	2393 (8.0/94.2)	.01
Stomach	1359 (4.3)	77 (4.0/5.7)	23 (3.9/1.7)	21 (5.3/1.5)	10 (3.7/0.7)	<10	<10	<10	1282 (4.3/94.3)	.70
High-risk										
Lung	7899 (24.9)	573 (29.8/7.3)	188 (32.1/2.4)	135 (33.8/1.7)	88 (32.5/1.1)	80 (27.6/1.0)	36 (25.5/0.5)	46 (19.3/0.6)	7326 (24.6/92.7)	<.0001
Lymphoma	1791 (5.6)	90 (4.7/5.0)	22 (3.8/1.2)	16 (4.0/0.9)	13 (4.8/0.7)	12 (4.1/0.7)	<10	19 (8.0/1.1)	1701 (5.7/95.0)	.04
Gynecologic^c^	3191 (10.0)	231 (12.0/7.2)	63 (10.8/2.0)	40 (10.0/1.3)	34 (12.6/1.1)	36 (12.4/1.1)	24 (17.0/0.8)	34 (14.3/1.1)	2960 (9.9/92.8)	.003
Bladder	1915 (6.0)	110 (5.7/5.7)	35 (6.0/1.8)	21 (5.3/1.1)	20 (7.4/1.0)	20 (6.9/1.0)	<10	13 (5.5/0.7)	1805 (6.1/94.3)	.11
Testicular	296 (0.9)	16 (0.8/5.4)	4 (0.7/1.4)	4 (1.0/1.4)	4 (1.5/1.4)	<10	<10	<10	280 (0.9/94.6)	.92
Renal cell carcinoma	1240 (3.9)	83 (4.3/6.7)	34 (5.8/2.7)	13 (3.3/1.0)	14 (5.2/1.1)	<10	<10	<10	1157 (3.9/93.3)	.38
Other cancer types										
Colorectal	5358 (16.9)	350 (18.2/6.5)	100 (17.1/1.9)	66 (16.5/1.2)	37 (13.7/0.7)	56 (19.3/1.0)	24 (17.0/0.4)	67 (28.2/1.3)	5008 (16.8/93.5)	.0001
Breast	4381 (13.8)	223 (11.6/5.1)	69 (11.8/1.6)	36 (9.0/0.8)	25 (9.2/0.6)	40 (13.8/0.9)	20 (14.2/0.5)	33 (13.9/0.8)	4158 (13.9/94.9)	.0001
Prostate	2666 (8.4)	148 (7.7/5.6)	43 (7.3/1.6)	28 (7.0/1.1)	23 (8.5/0.9)	26 (9.0/1.0)	<10	24 (10.1/0.9)	2518 (8.4/94.4)	.0001
Other^d^	10,112 (31.8)	550 (28.6/5.4)	150 (25.6/1.5)	104 (26.1/1.0)	65 (24.0/0.6)	96 (33.1/0.9)	45 (31.9/0.4)	90 (37.8/0.9)	9562 (32.0/94.6)	<.0001
Metastatic vs nonmetastatic disease, *n* (%)^e^										
Metastatic disease (C77∗-C80∗)	21,994 (70.9)	1406 (75.6/6.4)	419 (73.3/1.9)	305 (79.4/1.4)	212 (80.9/1.0)	211 (75.4/1.0)	104 (76.5/0.5)	155 (68.9/0.7)	20,588 (70.6/93.6)	.0003
Nonmetastatic disease	8492 (27.4)	421 (22.7/5.0)	141 (24.7/1.7)	74 (19.3/0.9)	49 (18.7/0.6)	64 (22.9/0.8)	29 (21.3/0.3)	64 (28.4/0.8)	8071 (27.7/95.0)	
Unknown^f^	543 (1.8)	32 (1.7/5.9)	12 (2.1/2.2)	<10	<10	<10	<10	<10	511 (1.8/94.1)	
Baseline comorbidities										
CCI										
Mean (SD)	6.5 (3.2)	6.3 (3.2)	6.4 (3.3)	6.4 (3.2)	6.7 (3.2)	6.1 (3.2)	6.3 (3.2)	5.8 (3.2)	6.5 (3.2)	.004
Median (IQR)	8 (3-9)	8 (3-9)	8 (3-9)	8 (3-9)	8 (3-9)	8 (3-9)	8 (3-9)	8 (2-8)	8 (3-9)	
CCI 3 or 4	3130 (9.9)	185 (9.6/5.9)	42 (7.8/1.3)	46 (11.5/1.5)	27 (10.0/0.9)	29 (10.0/0.9)	14 (9.9/0.4)	27 (11.3/0.9)	2945 (9.9/94.1)	.06
CCI 5 or more	21,994 (69.2)	1291 (67.1/5.9)	404 (68.9/1.8)	265 (66.4/1.2)	195 (72.0/0.9)	191 (65.9/0.9)	93 (66.0/0.4)	143 (60.1/0.7)	20,703 (69.4/94.1)	
History of bleeding (≤24 mo before index date), all diagnoses, *n* (%)	4006 (12.6)	226 (11.7/5.6)	68 (11.6/1.7)	37 (9.3/0.9)	37 (13.7/0.9)	38 (13.1/0.9)	20 (14.2/0.5)	26 (10.9/0.6)	3780 (12.7/94.4)	.56
History of bleeding (≤24 mo before index date), principal diagnosis, *n* (%)	1305 (4.1)	76 (4.0/5.8)	31 (5.3/2.4)	14 (3.5/1.1)	13 (4.8/1.0)	10 (3.5/0.8)	<10	<10	1229 (4.1/94.2)	.26
Recent history of bleeding (≤3 mo before index date), principal diagnosis, *n* (%)	659 (2.1)	28 (1.5/4.2)	14 (2.4/2.1)	<10	<10	<10	<10	<10	631 (2.1/95.8)	.24
Comorbidities, *n* (%)										
Moderate to severe renal disease^g^	1421 (4.5)	69 (3.6/4.9)	23 (3.9/1.6)	15 (3.8/1.1)	11 (4.1/0.8)	10 (3.5/0.7)	<10	<10	1352 (4.5/95.1)	.29
Pulmonary disease	3731 (11.7)	224 (11.6/6.0)	79 (13.5/2.1)	50 (12.5/1.3)	28 (10.3/0.8)	29 (10.0/0.8)	14 (9.9/0.4)	24 (10.1/0.6)	3507 (11.8/94.0)	.62
Hypertension	11,343 (35.7)	603 (31.3/5.3)	185 (31.6/1.6)	116 (29.1/1.0)	83 (30.6/0.7)	91 (31.4/0.8)	54 (38.3/0.5)	74 (31.1/0.7)	10,740 (36.0/94.7)	.0004
Cerebrovascular disease	1452 (4.6)	81 (4.2/5.6)	27 (4.6/1.9)	18 (4.5/1.2)	13 (4.8/0.9)	10 (3.5/0.7)	<10	<10	1371 (4.6/94.4)	.73
Diabetes	5213 (16.4)	283 (14.7/5.4)	86 (14.7/1.6)	53 (13.3/1.0)	50 (18.5/1.0)	47 (16.2/0.9)	17 (12.1/0.3)	30 (12.6/0.6)	4930 (16.5/94.6)	.17
Obesity	3724 (11.7)	234 (12.16/6.3)	63 (10.8/1.7)	33 (8.3/0.9)	37 (13.7/1.0)	40 (13.8/1.1)	21 (14.9/0.6)	40 (16.8/1.1)	3490 (11.7/93.7)	.039
Anemia	9619 (30.3)	479 (24.9/5.0)	165 (28.2/1.7)	100 (25.1/1.0)	66 (24.4/0.7)	65 (22.4/0.7)	27 (19.2/0.3)	56 (23.5/0.6)	9140 (30.6/95.0)	<.0001
Recent history of falls	649 (2.0)	31 (1.6/4.8)	14 (2.4/2.2)	<10	<10	<10	<10	<10	618 (2.1/95.2)	.64
Concomitant antiplatelet agent (at index date ± 90 d), *n* (%)	6448 (20.3)	341 (17.7/5.3)	118 (20.1/1.8)	68 (17.0/1.1)	51 (18.8/0.8)	43 (14.8/0.7)	26 (18.4/0.4)	35 (14.7/0.5)	6107 (20.5/94.7)	.015
LMWH treatment duration (mo)										
Mean (SD)	7.01 (9.2)	11.8 (13.1)	5.4 (8.0)	7.4 (9.1)	9.0 (9.2)	13.3 (9.8)	19.4 (11.0)	32.0 (14.8)	6.7 (8.8)	<.0001
Median (IQR)	3.8 (1.4-8.5)	7.0 (2.6-16.1)	2.3 (1.0-6.4)	3.9 (2.2-9.0)	6.2 (4.2-10.0)	11.1 (8.0-15.0)	17.1 (14.3-22.4)	31.7 (23.1-42.1)	3.7 (1.31-8.1)	
Time from index date to first VTE recurrence (mo)										
Mean (SD)	7.2 (10.2)	7.2 (10.2)	0.4 (0.3)	1.9 (0.6)	4.3 (0.8)	8.7 (1.8)	14.5 (1.7)	29.9 (10.2)	NA	<.0001
Median (IQR)	2.8 (0.7-9.4)	2.8 (0.2-9.4)	0.4 (0.2-0.7)	1.8 (1.4-2.3)	4.1 (3.6-5.0)	8.5 (7.2-10.2)	14.1 (13.1-16.1)	27.0 (21.8-35.8)	NA	
Follow-up time (mo)^h^										
Mean (SD)	12.9 (15.5)	15.5 (15.6)	9.5 (13.3)	10.2 (12.3)	12.2 (12.9)	16.8 (12.4)	22.9 (11.6)	37.1 (13.1)	12.7 (15.4)	<.0001
Median (IQR)	6.7 (2.3-16.5)	9.8 (4.2-21.6)	4.3 (1.5-10.5)	5.3 (3.0-11.7)	7.2 (5.2-12.7)	12.4 (9.4-18.1)	18.5 (15.6-25.0)	33.7 (25.8-45.8)	6.6 (2.2;16.2)	
Death during follow-up, *n* (%)	18,185 (57.2)	1179 (61.3)	379 (64.7)	267 (66.9)	172 (63.5)	172 (59.3)	83 (58.9)	106 (44.5)	17,006 (57.0)	<.0001

For cells with two percentages – format: n (%/%) – the second percentage is the proportion of patients with or without the event during the specified timeframe among patients with the given baseline characteristic. All other percentages are proportions of patients with the given characteristic among patients with or without the event during the specified timeframe. Data are not shown for patients who switched to a vitamin K antagonist because the number of patients was <10.

CCI, Charlson Comorbidity Index; DVT, deep vein thrombosis; LMWH, low-molecular-weight heparin; NA, not applicable; PE, pulmonary embolism; Q, quintile; VTE, venous thromboembolism; ∗, all ICD-10 codes which start with the mentioned syntax (so, all ICD-10 codes starting with C77… until all ICD-10 codes starting with C80 were considered).

a Patients with recurrent VTE vs patients with no VTE recurrence: chi-squared test for categorical variables and ANOVA for continuous variables.

b Based on the area of residence at the time of the index VTE event.

c Gynecologic cancers included malignant neoplasms of the vulva, vagina, cervix uteri, corpus uteri, uterus (part unspecified), ovary, placenta, and other or unspecified female genital organs.

d Excluding colorectal, breast, and prostate cancer.

e Percentages were computed among patients with recorded information on metastatic disease status. Patients who developed metastatic disease >30 days after the index date were excluded from the analysis.

f International Classification of Diseases 10th revision information missing.

g International Classification of Diseases 10th revision: I120, I131, N032-N037, N052-N057, N18, N19, N250, Z490, Z491, Z492, Z940, or Z992; or *Classification Commune des Actes Médicaux* procedure codes for dialysis: JVJB001 or JVJB002 [29].

h From the index date until censoring or end of follow-up.

### 3.2 Patients who experienced VTE recurrence

A total of 1925 patients (6.1%) had a recurrent VTE event during the study period (Table 1 and Supplementary Table S1). One hundred thirty-nine of these patients also experienced bleeding: 78 after VTE recurrence and 61 before VTE recurrence. Nearly two-thirds of patients (65.2%) first experienced VTE recurrence within 6 months after the index date, with the greatest proportion of events (30.4%) occurring within 1 month after the index date. The median (IQR) time from the index date to the first recurrent VTE event was 2.8 (0.7-9.4) months. Generally, the number of VTE events declined during the course of follow-up.

The mean (SD) age of patients who experienced VTE recurrence was 64.2 (13.2) years, and 51.0% of those patients were male. Compared with patients with VTE recurrence, a lower percentage of patients without VTE recurrence had lung cancer (24.5% vs 29.8%; *P* < .0001) or colorectal cancer (16.8% vs 18.2%; *P* = .0001). By contrast, the percentage of patients without VTE recurrence who had breast cancer was higher than the corresponding percentage of patients with VTE recurrence (13.9% vs 11.6%; *P* = .0001). Compared with patients with VTE recurrence, a lower percentage of patients without VTE recurrence had metastatic disease (70.6% vs 75.6%; *P* = .0003 for the distribution).

### 3.3 Patients with bleeding leading to hospitalization

A total of 1804 patients (5.7%) had a bleeding event leading to hospitalization (Table 2 and Supplementary Table S1). Six hundred ninety-nine patients (38.7%) had gastrointestinal bleeding, 229 (12.7%) had intracranial bleeding, and 65 (3.6%) had abnormal uterine bleeding. Other types of bleeding included uterine and vaginal, intraocular, otorrhagia, pericardial, respiratory, and intra-articular. Just under two-thirds of patients (62.6%) first experienced bleeding within 6 months after the index date. The median (IQR) time to the first bleeding event was 3.7 (1.0-9.7) months. As with VTE recurrence, bleeding events peaked in the first month after the index date (when 25.6% of events occurred) and generally declined over time.

**Table 2:** Characteristics of patients with and without a bleeding event as a principal diagnosis.

Characteristic	LMWH overall (*N* = 31,771)	Bleeding event (principal diagnosis) during follow-up								
		Anytime during follow-up (*n* = 1804, 5.7%)	0-1 mo (*n* = 462, 25.6%)	>1-3 mo (*n* = 354, 19.6%)	>3-6 mo (*n* = 313, 17.4%)	>6-12 mo (*n* = 309, 17.1%)	>12-18 mo (*n* = 142, 7.9%)	>18 mo (*n* = 224, 12.4%)	No bleeding event (*n* = 29,967, 94.3%)	*P* value^a^
Type of bleeding, *n* (%)^b^										
Gastrointestinal	699 (2.2)	699 (38.8/100)	171 (37.0/24.5)	150 (42.4/21.5)	112 (35.8/16.0)	117 (37.9/16.7)	57 (40.1/8.2)	92 (41.1/13.2)	-	-
Intracranial	229 (0.7)	229 (12.7/100)	41 (8.9/17.9)	40 (11.3/17.5)	51 (16.3/22.3)	44 (14.2/19.2)	14 (9.9/6.1)	39 (17.4/17.0)	-	
Abnormal uterine	65 (0.2)	65 (3.6/100)	17 (3.9/26.2)	15 (4.2/23.1)	11 (3.5/16.9)	5 (1.6/7.7)	7 (4.9/10.8)	10 (4.5/15.4)	-	
Other^c^	887 (2.8)	887 (49.2/100)	252 (54.6/28.4)	166 (46.9/18.7)	154 (49.2/17.4)	150 (48.5/16.9)	71 (50.0/8.0)	94 (42.0/10.6)	-	
Age at index date (y)										
Mean (SD)	66.3 (13.2)	67.2 (13.0)	67.5 (12.8)	68.0 (12.9)	66.8 (13.0)	66.6 (13.9)	66.5 (13.6)	66.8 (11.9)	66.3 (13.2)	.040
Median (IQR)	67.0 (58.4-75.9)	67.7 (59.6-77.1)	67.7 (59.8-77.9)	68.6 (60.0-77.3)	67.2 (60.0-76.4)	68.0 (58.8-76.8)	68.2 (60.2-75.6)	66.9 (59.0-76.6)	67.0 (58.3-75.8)	
Male, *n* (%)	16,189 (51.0)	1042 (57.8/6.4)	272 (58.9/1.7)	204 (57.6/1.3)	177 (56.6/1.1)	177 (57.3/1.1)	81 (57.0/0.5)	131 (58.5/0.8)	15,147 (50.6/93.6)	<.0001
Deprivation index, *n* (%)^d^										
Q4	11,385 (35.8)	623 (34.5/5.5)	152 (32.9/1.3)	122 (34.5/1.1)	114 (36.4/1.0)	91 (29.5/0.8)	62 (43.7/0.5)	82 (36.6/0.7)	10,762 (35.9/94.5)	.19
Q5	1657 (5.2)	94 (5.2/5.7)	18 (3.9/1.1)	25 (7.1/1.5)	14 (4.5/0.8)	22 (7.1/1.3)	<10	<10	1563 (5.2/94.3)	
Type of VTE, *n* (%)										
DVT alone	13,109 (41.3)	732 (40.6/5.6)	176 (38.1/1.3)	144 (40.7/1.1)	130 (41.5/1.0)	130 (42.1/1.0)	64 (45.1/0.5)	88 (39.3/0.7)	12,377 (41.3/94.4)	.69
PE (with or without DVT)	18,662 (58.7)	1072 (59.4/5.7)	286 (61.9/1.5)	210 (59.3/1.1)	183 (58.5/1.0)	179 (57.9/1.0)	78 (54.9/0.4)	136 (60.7/0.7)	17,590 (58.7/94.3)	
Rate of bleeding by cancer type, *n* (%)										
Very high-risk										
Brain	1149 (3.6)	76 (4.2/6.6)	15 (3.3/1.3)	14 (4.0/1.2)	14 (4.5/1.2)	17 (5.5/1.5)	<10	11 (4.9/1.0)	1073 (3.6/93.4)	.35
Pancreatic	2541 (8.0)	137 (7.6/5.4)	42 (9.1/1.7)	28 (7.9/1.1)	15 (4.8/0.6)	22 (7.1/0.9)	14 (9.9/0.6)	16 (7.1/0.6)	2404 (8.0/94.6)	.81
Stomach	1359 (4.3)	132 (7.3/9.7)	40 (8.7/2.9)	29 (8.2/2.1)	23 (7.4/1.7)	24 (7.8/1.8)	5 (3.5/0.4)	11 (4.9/0.8)	1227 (4.1/90.3)	<.0001
High-risk										
Lung	7899 (24.9)	392 (21.7/5.0)	98 (21.2/1.2)	75 (21.2/0.9)	71 (22.7/0.9)	76 (24.6/1.0)	32 (22.5/0.4)	40 (17.9/0.5)	7507 (25.1/95.0)	.01
Lymphoma	1791 (5.6)	84 (4.7/4.7)	21 (4.6/1.2)	14 (4.0/0.8)	18 (5.8/1.0)	10 (3.2/0.6)	10 (7.0/0.6)	11 (4.9/0.6)	1707 (5.7/95.3)	.19
Gynecologic^e^	3191 (10.0)	192 (10.6/6.0)	46 (10.0/1.4)	33 (9.3/1.0)	34 (10.9/1.1)	30 (9.7/0.9)	21 (14.8/0.7)	28 (12.5/0.9)	2999 (10.0/94.0)	.28
Bladder	1915 (6.0)	176 (9.8/9.2)	42 (9.1/2.2)	41 (11.6/2.1)	35 (11.2/1.8)	27 (8.7/1.4)	11 (7.8/0.6)	20 (8.9/1.0)	1739 (5.8/90.8)	<.0001
Testicular	296 (0.9)	11 (0.6/3.7)	<10	<10	<10	<10	<10	<10	285 (1.0/96.3)	.52
Renal cell carcinoma	1240 (3.9)	98 (5.4/7.9)	27 (5.8/2.2)	18 (5.1/1.5)	22 (7.0/1.8)	15 (4.9/1.2)	<10	<10	1142 (3.8/92.1)	.007
Other cancer types										
Colorectal	5358 (16.9)	325 (18.0/6.1)	65 (14.1/1.2)	58 (16.4/1.1)	56 (17.9/1.0)	58 (18.8/1.1)	30 (21.1/0.6)	58 (25.9/1.1)	5033 (16.8/93.9)	.002
Breast	4381 (13.8)	179 (9.9/4.1)	40 (8.7/0.9)	36 (10.2/0.8)	30 (9.6/0.7)	29 (9.4/0.7)	13 (9.2/0.3)	31 (13.8/0.7)	4202 (14.0/95.9)	.002
Prostate	2666 (8.4)	227 (12.6/8.5)	70 (15.2/2.6)	37 (10.5/1.4)	39 (12.5/1.5)	45 (14.6/1.7)	15 (10.6/0.6)	21 (9.4/0.8)	2439 (8.1/91.5)	.002
Other^f^	10,112 (31.8)	595 (33.0/5.9)	136 (29.4/1.3)	117 (33.1/1.2)	104 (33.3/1.0)	112 (36.3/1.1)	50 (35.2/0.5)	76 (33.9/0.8)	9517 (31.8/94.1)	.4
Metastatic vs nonmetastatic disease, *n* (%)^g^										
Metastatic disease (C77∗-C80∗)	21,994 (70.9)	1305 (74.9/5.9)	326 (72.6/1.5)	260 (74.9/1.2)	238 (78.0/1.1)	238 (79.6/1.1)	101 (74.3/0.5)	142 (68.6/0.6)	20,689 (70.6/94.1)	.002
Nonmetastatic disease	8492 (27.4)	419 (24.0/4.9)	117 (26.1/1.4)	85 (24.5/1.0)	64 (21.0/0.8)	58 (19.4/0.7)	34 (25.0/0.4)	61 (29.5/0.7)	8073 (27.6/95.1)	
Unknown^h^	543 (1.8)	19 (1.1/3.5)	<10	<10	<10	<10	<10	<10	524 (1.8/96.5)	
Baseline comorbidities										
CCI										
Mean (SD)	6.5 (3.2)	6.7 (3.3)	6.8 (3.2)	7.0 (3.2)	6.7 (3.3)	6.9 (3.3)	6.21 (3.3)	5.9 (3.4)	6.4 (3.2)	<.0001
Median (IQR)	8 (3-9)	8 (3-9)	8 (3-9)	8 (4-9)	8 (3-9)	8 (3-9)	8 (3-9)	6 (3-8)	8 (3-9)	
CCI 3 or 4	3130 (9.9)	181 (10.0/5.8)	38 (8.2/1.2)	36 (10.2/1.2)	28 (9.0/0.9)	33 (10.7/1.1)	12 (8.5/0.4)	34 (15.2/1.1)	2949 (9.8/94.2)	.0001
CCI 5 or more	21,994 (69.2)	1296 (71.8/5.9)	342 (74.0/1.6)	267 (75.4/1.2)	226 (72.2/1.0)	230 (74.4/1.0)	95 (66.9/0.4)	136 (60.7/0.6)	20,698 (69.1/94.1)	
History of bleeding (≤24 mo before index date), all diagnoses, *n* (%)	4006 (12.6)	406 (22.5/10.1)	145 (31.4/3.6)	84 (23.7/2.1)	64 (20.5/1.6)	64 (20.7/1.6)	11 (7.8/0.3)	38 (17.0/0.9)	3600 (12.0/89.9)	<.0001
History of bleeding (≤24 mo before index date), principal diagnosis, *n* (%)	1305 (4.1)	159 (8.8/12.2)	65 (14.1/5.0)	31 (8.8/2.4)	23 (7.4/1.8)	25 (8.1/1.9)	3 (2.1/0.2)	12 (5.4/0.9)	1146 (3.8/87.8)	<.0001
Recent history of bleeding (≤3 mo before index date), principal diagnosis, *n* (%)	659 (2.1)	93 (5.2/14.1)	39 (8.44/5.9)	24 (6.8/3.6)	11 (3.5/1.7)	11 (3.6/1.7)	3 (2.1/0.5)	5 (2.2/0.8)	566 (1.9/85.9)	<.0001
Comorbidities, *n* (%)										
Moderate to severe renal disease^i^	1421 (4.5)	119 (6.6/8.4)	29 (6.3/2.0)	27 (7.6/1.9)	15 (4.8/1.1)	26 (8.4/1.8)	11 (7.8/0.8)	11 (4.9/0.8)	1302 (4.3/91.6)	<.0001
Pulmonary disease	3731 (11.7)	228 (12.6/6.1)	57 (12.3/1.5)	53 (15.0/1.4)	36 (11.5/1.0)	39 (12.6/1.0)	16 (11.3/0.4)	27 (12.1/0.7)	3503 (11.7/93.9)	
Hypertension	11,343 (35.7)	707 (39.2/6.2)	185 (40.0/1.6)	133 (37.6/1.2)	123 (39.3/1.1)	128 (41.4/1.1)	50 (35.2/0.4)	88 (39.3/0.8)	10,636 (35.5/93.8)	.019
Cerebrovascular disease	1452 (4.6)	107 (5.9/7.4)	32 (6.9/2.2)	16 (4.5/1.1)	24 (7.7/1.7)	15 (4.9/1.0)	<10	13 (5.8/0.9)	1345 (4.5/92.6)	.04
Diabetes	5213 (16.4)	342 (19.0/6.6)	90 (19.5/1.7)	78 (22.0/1.5)	55 (17.6/1.1)	54 (17.5/1.0)	27 (19.0/0.5)	38 (17.0/0.7)	4871 (16.3/93.4)	.03
Obesity	3724 (11.7)	231 (12.8/6.2)	63 (13.6/1.7)	50 (14.1/1.3)	34 (10.9/0.9)	40 (12.9/1.1)	14 (9.9/0.4)	30 (13.4/0.8)	3493 (11.7/93.8)	.48
Anemia	9619 (30.3)	625 (34.7/6.5)	190 (41.1/2.0)	129 (36.4/1.3)	103 (32.9/1.1)	102 (33.0/1.1)	35 (24.7/0.4)	66 (29.5/0.7)	8994 (30.0/93.5)	<.0001
Recent history of falls	649 (2.0)	30 (1.7/4.6)	<10	<10	<10	<10	<10	<10	619 (2.1/95.4)	.27
Concomitant antiplatelet agent (at index date ± 90 d), *n* (%)	6448 (20.3)	445 (24.7/6.9)	128 (27.7/2.0)	81 (22.9/1.3)	83 (26.5/1.3)	71 (23.0/1.1)	28 (19.7/0.4)	54 (24.1/0.8)	6003 (20.0/93.1)	<.0001
LMWH treatment duration (mo)										
Mean (SD)	7.0 (9.2)	9.4 (10.9)	5.0 (7.7)	6.0 (6.9)	7.8 (7.9)	10.6 (9.2)	14.5 (10.1)	21.3 (16.4)	6.9 (9.0)	<.0001
Median (IQR)	3.8 (1.4-8.5)	5.7 (2.0-12.0)	1.9 (1.0;6.0)	3.3 (2-7.2)	5.8 (4.0-7.9)	8.8 (6.0-12.0)	14.1 (6.8-17.9)	21.6 (6.1-31.8)	3.7 (1.3-8.3)	
Time from index date to first bleeding event (mo)										
Mean (SD)	7.6 (10.4)	7.6 (10.4)	0.4 (0.3)	1.9 (0.6)	4.4 (0.9)	8.4 (1.8)	14.7 (1.7)	30.4 (11.3)	NA	<.0001
Median (IQR)	3.7 (1.0-9.7)	3.7 (1.0-9.7)	0.4 (0.2-0.7)	1.8 (1.4-2.5)	4.4 (3.7-5.1)	8.2 (7.0-9.9)	14.6 (13.3-16.2)	27.6 (21.1-37.2)	NA	
Follow-up time (mo)^j^										
Mean (SD)	12.9 (15.5)	15.7 (15.7)	9.1 (13.4)	9.4 (11.7)	12.7 (12.1)	15.7 (11.6)	22.9 (10.5)	38.6 (14.4)	12.7 (15.4)	<.0001
Median (IQR)	6.7 (2.3-16.5)	10.0 (4.4-22.1)	3.7 (1.4-10.1)	4.8 (2.7-10.8)	7.6 (5.5-14.5)	11.8 (9.0-16.4)	19.4 (16.0-25.0)	34.7 (26.9-46.9)	6.6 (2.2-16.2)	
Death during follow-up, *n* (%)	18,185 (57.2)	1289 (71.5)	341 (73.8)	274 (77.4)	232 (74.1)	236 (76.4)	91 (64.1)	115 (51.3)	16,896 (56.4)	<.0001

For cells with two percentages – format: n (%/%) – the second percentage is the proportion of patients with or without the event during the specified timeframe among patients with the given baseline characteristic. All other percentages are proportions of patients with the given characteristic among patients with or without the event during the specified timeframe. Data are not shown for patients who switched to a vitamin K antagonist because the number of patients was <10.

CCI, Charlson comorbidity index; DVT, deep vein thrombosis; LMWH, low-molecular-weight heparin; NA, not applicable; PE, pulmonary embolism; Q, quintile; VTE, venous; ∗, all ICD-10 codes which start with the mentioned syntax (so, all ICD-10 codes starting with C77… until all ICD-10 codes starting with C80 were considered).

a Patients with a bleeding event as a principal diagnosis vs patients with no bleeding event: chi-squared test for categorical variables and ANOVA for continuous variables.

b Individual patients could have more than 1 type of bleeding.

c Other bleeding sites included uterine and vaginal, intraocular, otorrhagia, pericardial, respiratory, and intra-articular.

d Based on the area of residence at the time of the index VTE event.

e Gynecologic cancers included malignant neoplasms of the vulva, vagina, cervix uteri, corpus uteri, uterus (part unspecified), ovary, placenta, and other or unspecified female genital organs.

f Excluding colorectal, breast, and prostate cancer.

g Percentages were computed among patients with recorded information on metastatic disease status. Patients who developed metastatic disease >30 days after the index date were excluded from the analysis.

h International Classification of Diseases 10th revision information missing.

i International Classification of Diseases 10th revision: I120, I131, N032-N037, N052-N057, N18, N19, N250, Z490, Z491, Z492, Z940, or Z992; or *Classification Commune des Actes Médicaux* procedure codes for dialysis: JVJB001 or JVJB002 [29].

j From the index date until censoring or end of follow-up.

Compared with patients with bleeding, lower percentages of patients without bleeding had colorectal cancer (16.8% vs 18.0%; *P* = .002), prostate cancer (8.1% vs 12.6%; *P* = .002), or stomach cancer (4.1% vs 7.3%; *P* < .0001). By contrast, the percentages of patients without bleeding who had lung cancer (25.1% vs 21.7%; *P* = .01) and breast cancer (14.0% vs 9.9%; *P* = .002) were higher than the corresponding percentages of patients with bleeding. Of patients with bleeding, 74.9% had metastatic disease, a higher percentage than for patients without bleeding (70.6%; *P* = .002 for the distribution).

### 3.4 AC switching within 1 month after VTE recurrence

Of 1925 patients with a recurrent VTE event, 370 (19.2%) subsequently switched to a different AC: 179 to a DOAC, 83 to a VKA, and 108 to another parenteral AC. One hundred forty-three patients (7.4%) switched AC within 1 month after VTE recurrence: 62 (43.4%) to a DOAC, 28 (19.6%) to a VKA, and 50 (35.0%) to a parenteral AC (Supplementary Table S2). Of the 62 patients who switched to a DOAC, <10 switched to apixaban 2.5 mg, 16 to apixaban 5 mg, 14 to rivaroxaban 15 mg, 11 to rivaroxaban 20 mg, and 20 to successive 15 mg and 20 mg doses of rivaroxaban.

Compared with patients who switched AC within 1 month after VTE recurrence, those who did not switch were more likely to have lung cancer (30.4%; *P* = .03) and metastatic disease (77.2%; *P* < .0001 for the distribution) and less likely to have colorectal cancer (17.6%; *P* = .02).

Median (IQR) time to the first recurrent VTE event was 6.8 (1.6-17.0) months for patients who switched AC, ranging from 2.1 (0.7-6.8) months for those who switched to a parenteral AC to 14.8 (6.5-31.4) months for those who switched to a DOAC. For patients who did not switch AC, the median (IQR) time to first VTE recurrence was 2.7 (0.70-8.9) months.

### 3.5 Treatment outcomes in patients with an AC switch within 1 month after VTE recurrence

Nine of the 143 patients who switched AC within 1 month after their first recurrent VTE event (6.3%) had a second recurrent VTE event before AC switching. Of the remaining 134 patients, 4 (3.0%) had another recurrent VTE event within 3 months after their first recurrent VTE event, 6 (4.5%) within 6 months after their first recurrent VTE event, and 12 (9.0%) anytime during follow-up after their first recurrent VTE event. Three patients (2.2%) had a bleeding event and 13 (9.7%) died within 3 months after their first recurrent VTE event; 5 (3.7%) had a bleeding event and 26 (19.4%) died within 6 months after their first recurrent VTE event; and 9 (6.7%) had a bleeding event and 58 (43.3%) died anytime during follow-up after their first recurrent VTE event.

### 3.6 AC switching within 1 month after a bleeding event

A total of 159 patients (8.8%) switched AC after a bleeding event: 73 to a DOAC, 21 to a VKA, and 65 to another parenteral AC. Forty-two patients (2.3%) switched AC within 1 month after a bleeding event (Supplementary Table S3). Of these patients, 23 (54.8%) switched to a parenteral AC and 19 (45.2%) to an oral AC. Of those who switched to an oral AC, 13 (68.4%) switched to a DOAC. For patients who switched to apixaban or rivaroxaban, <10 patients received each different dose level. Fewer than 10 patients who experienced bleeding stopped receiving AC treatment.

Compared with patients who switched AC within 1 month after a bleeding event, a greater proportion of patients who did not switch AC had metastatic disease (75.1% vs 66.7%, *P* = .0003 for the distribution).

Median (IQR) time to first bleeding event was 3.3 (0.8-11.2) months overall for patients who switched AC, 6.5 (1.2-11.2) months for those who switched to a DOAC, 2.9 (1.2-13.6) months for those who switched to a parenteral AC, and 3.7 (1.0-9.7) months for patients who did not switch AC.

### 3.7 Summary of treatment patterns and clinical outcomes after VTE recurrence

**Figure 2:**
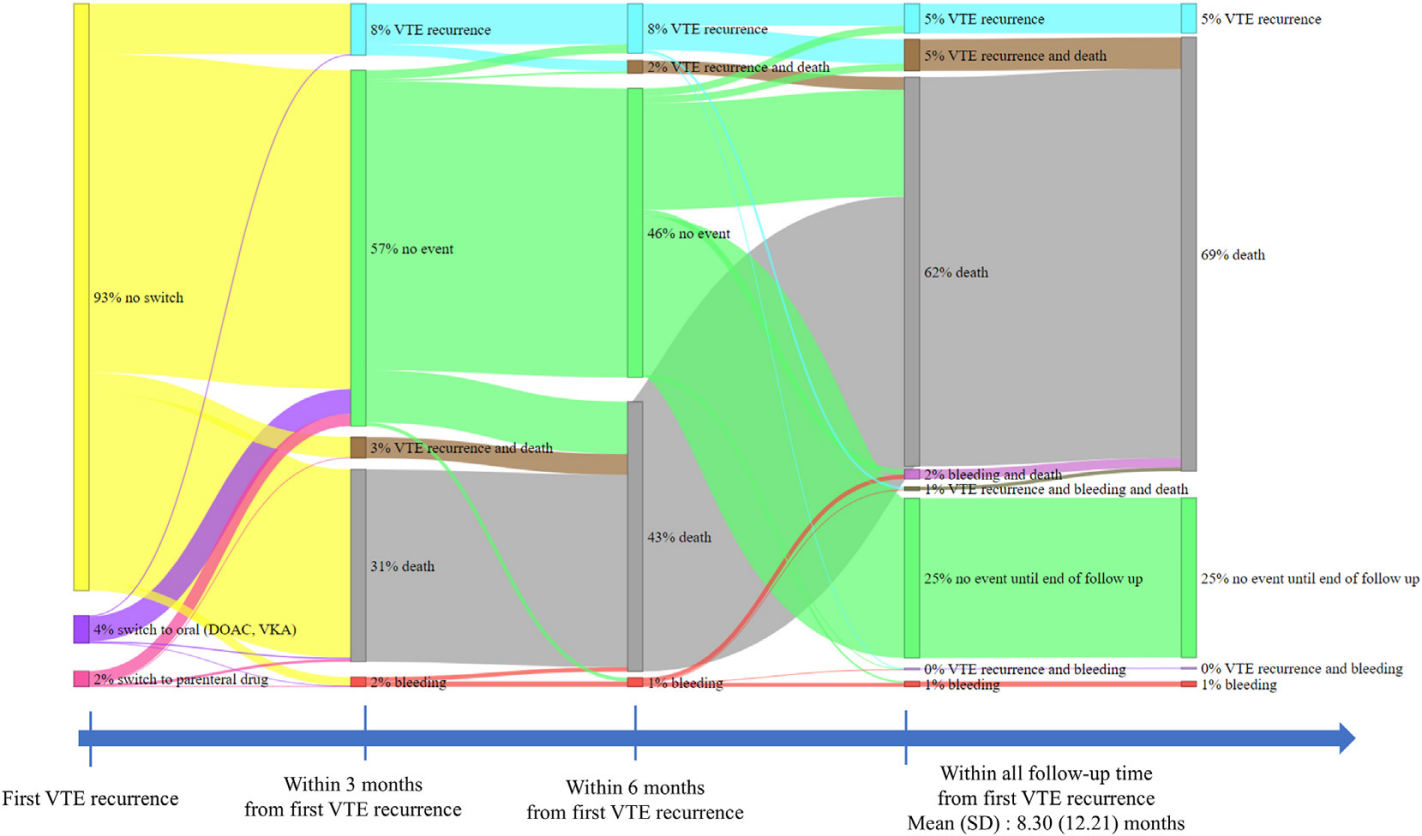
Sankey diagram showing treatment patterns and outcomes after venous thromboembolism (VTE) recurrence. Causes of death are not specified. DOAC, direct oral anticoagulant; VKA, vitamin K antagonist.

AC treatment patterns and clinical outcomes after a first recurrent VTE event are summarized in Figure 2. Notably, most patients who experienced VTE recurrence did not switch AC. Moreover, very few patients with VTE recurrence subsequently experienced a bleeding event, and the risk of a second recurrent VTE event was higher than the risk of bleeding. At 3 months, 31% of patients had died without experiencing further VTE recurrence, and 11% had experienced further VTE recurrence (3% experienced further VTE recurrence and died, and 8% experienced further VTE recurrence but survived). By the end of follow-up, 69% of patients had died.

## 4 Discussion

In this nationwide analysis of patient characteristics, secondary AC management, and treatment outcomes of patients with cancer who were treated with LMWH after an initial VTE event, more than 10% of patients experienced treatment failure, as defined by VTE recurrence or bleeding. Notably, recurrent VTE and bleeding events were most frequent in the first month after the initial VTE event. Compared with patients without VTE recurrence, those with VTE recurrence were more likely to have lung cancer or colorectal cancer. Patients with bleeding were more likely to have colorectal cancer, prostate cancer, or stomach cancer than patients without bleeding. Moreover, metastatic disease was more frequent in patients with VTE recurrence or bleeding than in patients without these events.

More than a third of patients who switched AC after VTE recurrence and approximately a quarter of patients who switched AC after bleeding did so within 1 month, likely reflecting clinical management of the event. Patients who switched to a DOAC within 1 month after VTE recurrence were more likely to receive a standard DOAC dose than a reduced dose. The picture was less clear for DOAC doses received after bleeding events, partly because of the modest number of patients who switched AC after a bleeding event. Compared with patients who did not switch AC within 1 month after VTE recurrence, those who switched AC were less likely to have lung cancer and more likely to have colorectal cancer. Patients who switched AC within 1 month after a recurrent VTE or bleeding event were less likely to have metastatic disease than patients who did not switch AC.

The overall rate of VTE recurrence in the present study (6.1%) was comparable with the rates in the Caravaggio randomized, open-label, noninferiority trial of apixaban (7.9% at 6 months in the LMWH [dalteparin] arm) and the PREDICARE prospective observational study of the LMWH drug tinzaparin (7.3% at 6 months) [31,32]. This was despite the duration of follow-up being longer in our study. The overall rate of VTE recurrence was also comparable with that in a prospective real-world evidence study (5.6% in those prescribed LMWH), which had a longer duration of follow-up than the present study [5]. The rate of VTE recurrence was lower than in the dalteparin arm of the French CASTA DIVA trial of rivaroxaban (10.1% at 3 months), although it should be noted that CASTA DIVA selected patients with a high risk of VTE recurrence and used a different definition of VTE that also included worsening of pulmonary or lower limb vascular obstruction [33].

Our patients with VTE recurrence had similar characteristics as other reported populations of patients with cancer who experienced VTE recurrence. For example, the mean age of patients who experienced VTE recurrence was 64.2 years in our study, similar to the age reported in the Caravaggio trial (65.5 years) [34]. Moreover, approximately half of patients with VTE recurrence were male, as reported in both Caravaggio and a real-world evidence study of patients with cancer in the UK who experienced VTE [3,34]. In our study, the median time to first VTE recurrence was 2.8 months, and just under a third of recurrent VTE events occurred in the first month after the initial VTE event, mirroring what was found in Caravaggio [31].

Preventing bleeding remains a major challenge in patients receiving AC treatment for cancer-associated VTE [6,35]. In the present study, the highest percentage of bleeding events (approximately 39%) were gastrointestinal, similar to other studies of patients with cancer who received AC treatment [[36], [37], [38]]. In agreement with recent meta-analyses of patients receiving ACs for cancer-associated VTE [18,19], we found that the rate of bleeding was lower than the rate of VTE recurrence, even in patients who had already experienced a first recurrent VTE event.

Because large numbers of recurrent VTE and bleeding events occurred within 1 month after the initial VTE event, it is important to consider the potential benefits and risks of different AC treatment options immediately after an initial VTE event. Choosing the best option is often challenging because many patients with cancer-associated VTE are frail. Moreover, the benefit-risk equation for AC treatment differs depending on cancer type and stage, with rates of VTE recurrence varying markedly between different types of cancer and between metastatic and nonmetastatic disease [34,39,40]. Physicians should take these and other patient characteristics into account when making AC prescribing decisions.

During the period covered by the present analysis, treatment guidelines recommended continuing LMWH at a higher dose in patients who experienced cancer-associated VTE recurrence. Accordingly, most patients who experienced VTE recurrence continued to receive LMWH. (We had no access to data on LMWH dose modifications, and so were unable to determine whether these patients received a higher dose.) However, the risk of further VTE recurrence was high. Optimal treatment pathways for managing cancer-related VTE recurrence are unclear because the evidence is limited on what to do when a patient experiences recurrence: whether to increase the LMWH dose or switch to a different AC. No randomized controlled trial has investigated this topic. Available evidence in favor of LMWH dose escalation comes from 2 modestly sized retrospective observational studies [21,41]; the findings of a third observational study were less conclusive [22]. Nonetheless, current American Society of Hematology and French treatment guidelines for cancer-associated VTE recommend increasing the LMWH dose to a supratherapeutic level in patients who experience VTE recurrence while receiving LMWH treatment [8,12].

Strengths of the present study include the large sample and long duration of follow-up. However, the statistical significance we report does not necessarily reflect clinical significance. A limitation of claims-based studies such as this is the possibility of data being misreported, misclassified, or miscoded. A further limitation of the present study is that we used only hospital records to identify cases of VTE recurrence and bleeding; events managed in an outpatient setting were not captured. This means that rates of these events may have been underestimated. Although most patients who experience VTE recurrence would be expected to be hospitalized, the same is not necessarily true for patients who experience bleeding. The observed pattern of bleeding sites – with high rates of intracranial and especially gastrointestinal bleeding – may reflect bias in favor of more severe events and may not be representative of all major and clinically relevant bleeding events. However, rates of recurrent VTE and bleeding events in the present study are comparable with rates reported from other studies, reinforcing the strength of our data. Another limitation is the nonavailability of data on other factors that potentially affect the risk of VTE recurrence or bleeding, such as renal impairment and thrombocytopenia. Nor could we properly analyze the influence of anticancer treatment because data were not available for cancer therapies administered in hospitals. Race/ethnicity is similarly unavailable in the SNDS database, which meant that we were unable to explore how study outcomes vary according to patient race/ethnicity. Data on in-hospital AC treatment were also unavailable to us, so we could not analyze this aspect either. Further, because we did not have access to patients’ medical charts, we were unable to estimate the sensitivity and positive predictive value of the VTE and bleeding definitions we used in the study. However, as noted above, our estimated rates of VTE recurrence were comparable with those from other studies [5,31,32].

Defining the index date as the date of the first LMWH reimbursement may have introduced immortal time bias because patients presumably received their first LMWH injections while still hospitalized, yet only patients who survived long enough to be discharged and claim an LMWH prescription were included in the analysis.

Although we compared crude characteristics of patients with vs without VTE recurrence and bleeding, the results are inevitably influenced by the characteristics of patients with index VTE events. Additional analyses, including risk factor analyses, could be conducted to further characterize the associations between patient characteristics and VTE recurrence and bleeding events. Finally, because relevant treatment guidelines were similar worldwide during the time period covered by our study, our findings are generalizable to other countries, albeit differences in reimbursement may have limited implementation of the guidelines in some countries.

In summary, our study highlights the risks of adverse outcomes (VTE recurrence and bleeding) in patients receiving LMWH for cancer-associated VTE and the challenge of preventing repeated VTE events in these patients. Currently, there is no clear strategy for managing patients with cancer who experience VTE recurrence while receiving ACs. To optimize secondary AC management in this patient population, there is an urgent need for prospective trials comparing different options, including dose increases and AC switching. Some of these options are currently being investigated in the REDUCE observational study (NCT05229471).
