# Supplementary material for: Occurrence and management of thrombosis recurrence and bleeding in low-molecular-weight heparin-treated patients with cancer-associated thrombosis: a French nationwide cohort study
Source: Res Pract Thromb Haemost. 2024 Nov 26;9(1):102642. doi: 10.1016/j.rpth.2024.102642 (PMC11759555; doi:10.1016/j.rpth.2024.102642)
Supplement: Supplemental Tables 1-3 [file mmc1.pdf]

Supplementary Materials

Supplementary Table 1. Characteristics of patients with recurrent VTE or bleeding at 0-6 months

Characteristic	VTE recurrence at 0-6 months (N=1,256)	Bleeding event (principal diagnosis) at 0-6 months (N=1,129)
Type of bleeding, n (%)[a]		
Gastrointestinal	-	433 (38.4)
Intracranial	-	132 (11.7)
Abnormal uterine	-	43 (3.8)
Other[b]	-	572 (50.7)
Age at index date (years)		
Mean (SD)	64.1 (13.5)	67.5 (12.9)
Median (IQR)	64.5 (55.2-73.6)	67.8 (59.8-77.4)
Male, n (%)	648 (51.6)	653 (57.8)
Deprivation index, n (%)[c]		
Q4	428 (34.1)	388 (34.4)
Q5	57 (4.5)	57 (5.1)
Type of VTE, n (%)		
DVT alone	534 (42.5)	450 (39.9)
PE (with or without DVT)	722 (57.5)	679 (60.1)
Rate of incident VTE by cancer type, n (%)		
Very high-risk		
Brain	47 (3.7)	43 (3.8)
Pancreatic	117 (9.3)	85 (7.5)
Stomach	54 (4.3)	92 (8.2)
High-risk		
Lung	411 (32.7)	244 (21.6)
Lymphoma	51 (4.1)	53 (4.7)
Gynecologic[d]	137 (10.9)	113 (10.0)
Bladder	76 (6.1)	118 (10.5)
Testicular	12 (1.0)	<10
Renal cell carcinoma	61 (4.9)	67 (5.9)
Other cancer types		
Colorectal	203 (16.2)	179 (15.9)
Breast	130 (10.4)	106 (9.4)
Prostate	94 (7.5)	146 (12.9)
Other[e]	319 (25.4)	357 (31.6)
Metastatic vs non-metastatic disease, n (%)[f]		
Metastatic disease (C77*-C80*)	936 (76.9)	824 (74.8)
Non-metastatic disease	264 (21.7)	266 (24.2)
Unknown[g]	18 (1.5)	11 (1.0)
Baseline comorbidities		
CCI		
Mean (SD)	6.5 (3.2)	6.8 (3.2)
Median (IQR)	8 (3-9)	8 (3-9)
CCI 3 or 4	115 (9.2)	102 (9.0)
CCI 5 or more	864 (68.8)	835 (74.0)
History of bleeding (≤24 months before index date), all diagnoses, n (%)	142 (11.3)	293 (26.0)
History of bleeding (≤24 months before index date), principal diagnosis, n (%)	58 (4.6)	119 (10.5)
Recent history of bleeding (≤3 months before index date), principal diagnosis, n (%)	22 (1.8)	74 (6.6)
Comorbidities, n (%)		
Moderate to severe renal disease[h]	49 (3.9)	71 (6.3)
Pulmonary disease	157 (12.5)	146 (12.9)
Hypertension	384 (30.6)	441 (39.1)
Cerebrovascular disease	58 (4.6)	72 (6.4)
Diabetes	189 (15.1)	223 (19.8)
Obesity	133 (10.6)	147 (13.0)
Anemia	331 (26.4)	422 (37.4)
Recent history of falls	22 (1.8)	23 (2.0)
Concomitant antiplatelet agent (at index date ± 90 days), n (%)	237 (18.9)	292 (25.9)
LMWH treatment duration (months)		
Mean (SD)	6.8 (8.8)	6.1 (7.6)
Median (IQR)	4.0 (1.6-8.3)	3.8 (1.5-7.1)
Time from index date to first VTE recurrence (months)		
Mean (SD)	1.7 (1.6)	-
Median (IQR)	1.2 (0.4-2.7)	-

Time from index date to first bleeding event (months)		
Mean (SD)	-	2.0 (1.7)
Median (IQR)	-	1.5 (0.5-3.3)
Follow-up time (months)[i]		
Mean (SD)	10.3 (12.9)	10.2 (12.6)
Median (IQR)	5.6 (2.8-11.5)	5.7 (2.7-11.6)
Death during follow-up, n (%)	818 (65.1)	847 (75.0)
Time to death (months)		
Mean (SD)	7.3 (8.2)	6.8 (7.4)
Median (IQR)	4.7 (2.5-8.9)	4.6 (2.2-8.1)

[a] Individual patients could have more than one type of bleeding

[b] Other bleeding sites included uterine and vaginal, intraocular, otorrhagia, pericardial, respiratory, and intra-articular

[c] Based on area of residence at the time of the index VTE event

[d] Gynecologic cancers included malignant neoplasms of the vulva, vagina, cervix uteri, corpus uteri, uterus (part unspecified), ovary, placenta, and other or unspecified female genital organs

[e] Excluding colorectal, breast, and prostate cancer

[f] Percentages were computed among patients with recorded information on metastatic disease status. Patients who developed metastatic disease more than 30 days after the index date were excluded from the analysis

[g] ICD-10 information missing

[h] ICD-10: I120, I131, N032-N037, N052-N057, N18, N19, N250, Z490, Z491, Z492, Z940, or Z992; or CCAM procedure codes for dialysis: JVJB001 or JVJB002 [29]

[i] From the index date until censoring or end of follow-up

Percentages are proportions of patients with the given characteristic among patients with or without the event during the specified timeframe. Data are not shown for patients who switched to a VKA because the number of patients was <10.

Abbreviations: CCI, Charlson comorbidity index; DVT, deep vein thrombosis; IQR, interquartile range; LMWH, low-molecular-weight heparin; Q, quintile; SD, standard deviation; VTE, venous thromboembolism

Supplementary Table 2. Characteristics of patients with and without an anticoagulant switch within 1 month after recurrent VTE

	All patients who had recurrent VTE (N=1,925)								
		Oral AC							
Characteristic	All patients who switched AC within 1 month out of 370 who switched AC during the entire follow-up (N=143, 38.7%)	Any oral AC (N=90, 62.9%)	DOAC (N=62, 43.4%)	VKA (N=28, 19.6%)	Parenteral AC (N=50, 35.0%)	No switch within 1 month (N=1,782, 92.6%)	P-value[a]	P-value[b]	P-value[c]
Patients who died, n (%)	0 (0)	0 (0)	0 (0)	0 (0)	0 (0)	1,179 (66.2)	-	-	-
Time to death from VTE recurrence (months)							-	-	-
Mean (SD)	NA	NA	NA	NA	NA	11.6 (11.4)	-	-	-
Median (IQR)	NA	NA	NA	NA	NA	7.7 (3.5-15.8)	-	-	-
Age at index date (years)									
Mean (SD)	64.7 (14.0)	67.0 (12.4)	66.4 (12.1)	68.4 (13.2)	60.8 (15.9)	64.2 (13.1)	0.05	0.03	0.7
Median (IQR)	66.1 (54.9-74.7)	68.0 (58.4- 75.8)	66.2 (58.4- 75.8)	70.1 (58.3-77.4)	59.5 (49.7-69.5)	64.8 (55.8-73.4)			
Index date, n (%)									
2013-2015	95 (66.4)	58 (64.4)	38 (61.3)	20 (71.4)	36 (72.0)	1084 (60.8)	0.29	0.23	0.19
2016-2018	48 (33.6)	32 (35.6)	24 (38.7)	<10	14 (28.0)	698 (39.2)			
Male, n (%)	81 (56.6)	48 (53.3)	36 (58.1)	12 (42.9)	31 (62.0)	901 (50.6)	0.21	0.25	0.16
Deprivation index, n (%)[d]									
Q4	50 (35.0)	32 (35.6)	22 (35.5)	10 (35.7)	16 (32.0)	609 (34.2)	0.99	0.98	-
Q5	<10	<10	<10	<10	<10	86 (4.8)			
Type of VTE, n (%)							0.001	0.77	0.82
DVT alone	58 (40.6)	34 (37.8)	15 (24.2)	19 (67.9)	22 (44.0)	706 (39.6)			
PE (with or without DVT)	85 (59.4)	56 (62.2)	47 (75.8)	<10	28 (56.0)	1076 (60.4)			
Risk of incident VTE by cancer type, n (%)									
Very high-risk	10 (7.0)	<10	<10	<10	<10	282 (15.8)	0.006	0.003	0.0006
Brain	<10	<10	<10	0 (0.0)	0 (0.0)	74 (4.2)	0.33	0.32	0.23
Pancreatic	<10	<10	<10	<10	<10	142 (8.0)	0.3	0.25	0.1
Stomach	<10	<10	0 (0.0)	<10	<10	75 (4.2)	0.35	0.26	0.1
High-risk	65 (45.5)	37 (41.1)	25 (40.3)	12 (42.9)	27 (54.0)	914 (51.3)			
Lung	31 (21.7)	12 (13.3)	<10	<10	18 (36.0)	542 (30.4)	0.005	0.002	0.03
Lymphoma	10 (7.0)	<10	<10	<10	<10	80 (4.5)	0.09	0.15	0.17
Gynecologic[e]	<10	<10	<10	<10	<10	225 (12.6)	0.03	0.01	0.003
Bladder	10 (7.0)	<10	<10	0 (0.0)	<10	100 (5.6)	0.26	0.72	0.49
Testicular	0 (0.0)	0 (0.0)	0 (0.0)	0 (0.0)	0 (0.0)	16 (0.9)	0.74	0.53	0.26
Renal cell carcinoma	<10	<10	<10	<10	<10	74 (4.2)	0.35	0.44	0.23
Other cancer types									
Colorectal	36 (25.2)	23 (25.6)	17 (27.4)	<10	12 (24.0)	314 (17.6)	0.15	0.09	0.02
Breast	15 (10.5)	<10	<10	<10	<10	208 (11.7)	0.79	0.63	0.67
Prostate	13 (9.1)	10 (11.1)	<10	<10	<10	135 (7.6)	0.34	0.29	0.51
Other[f]	45 (31.5)	31 (34.4)	24 (38.7)	<10	13 (26.0)	505 (28.3)	0.32	0.42	0.43
Metastatic vs non metastatic disease, n (%)[g]									

Metastatic disease (C77*-C80*)	77 (56.2)	39 (45.4)	28 (47.5)	11 (40.7)	36 (75.0)	1,329 (77.2)	<0.0001	<0.0001	<0.0001
Non-metastatic disease	55 (40.2)	43 (50.0)	29 (49.2)	14 (51.9)	11 (22.9)	366 (21.3)			
Unknown[h]	<10	<10	<10	<10	<10	27 (1.6)			
Baseline comorbidities (assessed in the 24 months before the index VTE event)									
CCI									
Mean (SD)	5.31 (3.3)	4.94 (3.2)	4.84 (3.2)	5.18 (3.3)	5.9 (3.4)	6.39 (3.2)	0.0003	0.0001	0.0001
Median (IQR)	4 (2-8)	3 (2-8)	3 (2-8)	4.5 (2-8)	8 (2-9)	8 (3-9)			
CCI 3 or 4, n (%)	17 (11.9)	12 (13.3)	10 (16.1)	<10	<10	168 (9.4)	0.002	0.0007	0.0002
CCI 5 or more, n (%)	74 (51.8)	43 (47.8)	28 (45.2)	15 (53.6)	29 (58.0)	1,217 (68.3)			
History of bleeding (≤24 months before index date), all diagnoses, n (%)	18 (12.6)	13 (14.4)	<10	<10	<10	208 (11.7)	0.85	0.67	0.74
History of bleeding (≤24 months before index date), principal diagnosis, n (%)	<10	<10	<10	<10	<10	72 (4.0)	0.82	0.69	0.46
Recent history of bleeding (≤3 months before index date), principal diagnosis, n (%)	0 (0.0)	0 (0.0)	0 (0.0)	0 (0.0)	0 (0.0)	28 (1.6)	0.53	0.33	0.13
Comorbidities, n (%)									
Moderate to severe renal disease[i]	<10	<10	<10	<10	<10	64 (3.6)	0.7	0.76	0.95
Pulmonary disease	12 (8.4)	<10	<10	<10	<10	212 (11.9)	0.16	0.09	0.21
Hypertension	46 (32.2)	33 (36.7)	21 (33.9)	12 (42.9)	13 (26.0)	557 (31.3)	0.46	0.4	0.82
Cerebrovascular disease	<10	<10	<10	<10	<10	74 (4.2)	0.88	0.81	0.67
Diabetes	27 (18.9)	16 (17.8)	13 (21.0)	<10	11 (22.0)	256 (14.4)	0.21	0.23	0.14
Obesity	29 (20.3)	18 (20.0)	15 (24.2)	<10	11 (22.0)	205 (11.5)	0.003	0.005	0.002
Anemia	36 (25.2)	27 (30.0)	16 (25.8)	11 (39.3)	<10	443 (24.9)	0.22	0.28	0.93
Recent history of falls	<10	<10	<10	<10	0 (0.0)	29 (1.6)	0.68	0.6	0.83
Concomitant antiplatelet agent (at index date ± 90 days), n (%)	30 (21.0)	16 (17.8)	12 (19.4)	<10	14 (28.0)	311 (17.5)	0.26	0.16	0.29
LMWH treatment duration (months)									
Mean (SD)	12.5 (13.9)	16.5 (15.3)	19.7 (16.1)	9.4 (10.6)	5.6 (7.4)	11.8 (13.0)	<0.0001	<0.0001	0.53
Median (IQR)	7.0 (2.1-17.1)	10.9 (4.5-25.6)	15.0 (6.9-31.6)	5.4 (1.4-12.8)	2.3 (0.9-7.6)	7.1 (2.7-16.0)			
Time to first VTE recurrence (months)									
Mean (SD)	12.2 (14.0)	16.2 (15.4)	19.4 (16.2)	9.0 (10.6)	5.3 (7.4)	6.8 (9.7)	<0.0001	<0.0001	<0.0001
Median (IQR)	6.8 (1.6-17.0)	10.4 (4.1-25.5)	14.8 (6.5-31.4)	5.1 (1.1-12.2)	2.1 (0.7-6.8)	2.7 (0.7-8.9)			
Follow-up time (months)[j]									
Mean (SD)	12.5 (13.9)	16.5 (15.3)	19.7 (16.1)	9.4 (10.6)	5.7 (7.4)	15.8 (15.7)	<0.0001	<0.0001	0.02
Median (IQR)	7.0 (2.1-17.1)	10.9 (4.5-25.6)	15.1 (7.0-31.7)	5.4 (1.4-12.8)	2.4 (1.0-7.7)	10.0 (4.3-21.8)			

[a] Comparison among patients who switched to a DOAC, switched to a VKA, switched to a parenteral AC, and did not switch: chi square test for categorical variables and ANOVA for continuous variables
[b] Comparison among patients who switched to an oral AC, switched to a parenteral AC, and did not switch: chi square test for categorical variables and ANOVA for continuous variables
[c] Comparison between patients who switched AC and those who did not switch: chi square test for categorical variables and ANOVA for continuous variables
[d] Based on area of residence at the time of the index VTE event

[e] Gynecologic cancers included malignant neoplasms of the vulva, vagina, cervix uteri, corpus uteri, uterus (part unspecified), ovary, placenta, and other and unspecified female genital organs
[f] Excluding colorectal, breast, and prostate cancer
[g] Percentages were computed among patients with recorded information on metastasis status. Patients who developed metastatic disease more than 30 days after the index date were excluded from the analysis
[h] ICD-10 information missing
[i] ICD-10: I120, I131, N032-N037, N052-N057, N18, N19, N250, Z490, Z491, Z492, Z940, or Z992; or CCAM procedure codes for dialysis: JVJB001 or JVJB002 [29]
[j] From the index date until censoring or end of follow-up
Percentages are proportions of patients with the given characteristic among patients with the specific AC switch event. Data are not shown for patients who switched to a VKA because the number of patients was <10.
Abbreviations: AC, anticoagulant; CCI, Charlson comorbidity index; DOAC, direct oral anticoagulant; DVT, deep vein thrombosis; ICD-10, International Classification of Diseases 10th revision; IQR, interquartile range; LMWH, low-molecular-weight heparin; PE, pulmonary embolism; Q, quintile; SD, standard deviation; VTE, venous thromboembolism

Supplementary Table 3. Characteristics of patients with and without an anticoagulant switch within 1 month after a bleeding event

	All patients who had a bleeding event (N=1,804)							
	All patients who switched AC within 1 month out of 159 who switched AC during the entire follow-up (N=42, 26.4%)	Oral AC						
Characteristic		Any oral AC (N=19, 45.2%)	DOAC (N=13, 31.0%)	Parenteral AC (N=23, 54.8%)	No switch within 1 month (N=1,762, 97.7%)	P-value[a]	P-value[b]	P-value[c]
Patients who died, n (%)	0.0	0.0	0.0	0.0	1,289 (73.2)	-	-	-
Time to death from VTE recurrence (months)								
Mean (SD)	NA	NA	NA	NA	11.0 (10.5)	-	-	-
Median (IQR)	NA	NA	NA	NA	7.6 (3.5-15.0)	-	-	-
Age at index date (years)								
Mean (SD)	66.9 (14.1)	69.8 (13.9)	69.4 (11.6)	64.4 (14.1)	67.2 (13.0)	0.59	0.4	0.87
Median (IQR)	68.7 (58.5-79.4)	73.1 (62.2-81.7)	69.8 (62.2-80.3)	67.7 (55.5-76.0)	67.7 (60.0-77.0)			
Index date, n (%)								
2013-2015	27 (64.3)	<10	<10	18 (78.3)	1109 (62.9)	0.03	0.12	0.86
2016-2018	15 (35.7)	10 (52.6)	<10	<10	653 (37.1)			
Male, n (%)	28 (66.7)	13 (68.4)	10 (76.9)	15 (65.2)	1014 (57.6)	0.45	0.49	0.24
Deprivation index, n (%)[d]								
Q4	12 (28.6)	<10	<10	<10	611 (34.7)	0.19	0.16	-
Q5	<10	0 (0.0)	0 (0.0)	<10	93 (5.3)			
Type of VTE, n (%)								
DVT alone	13 (31.0)	<10	<10	<10	719 (40.8)	0.41	0.44	0.20
PE (with or without DVT)	29 (69.1)	13 (68.4)	10 (76.9)	16 (69.6)	1,043 (59.2)			
Risk of incident bleeding, by cancer type, n (%)	<10	<10	<10	<10	332 (18.8)	0.001	0.0005	0.0002
Very high-risk								
Brain	0 (0.0)	0 (0.0)	0 (0.0)	0 (0.0)	76 (4.3)	0.60	0.39	0.17
Pancreatic	<10	<10	<10	<10	135 (7.7)	0.84	0.78	0.48
Stomach	<10	<10	0 (0.0)	0 (0.0)	131 (7.4)	0.30	0.37	0.21
High-risk	17 (40.5)	<10	<10	11 (47.5)	782 (44.4)			
Lung	<10	<10	<10	<10	386 (21.9)	0.52	0.43	0.24
Lymphoma	<10	<10	0 (0.0)	0 (0.0)	83 (4.7)	0.29	0.56	0.48
Gynecologic[e]	<10	0 (0.0)	0 (0.0)	<10	189 (10.7)	0.49	0.30	0.46
Bladder	<10	<10	<10	<10	171 (9.7)	0.88	0.86	0.63
Testicular	0 (0.0)	0 (0.0)	0 (0.0)	0 (0.0)	11 (0.6)	0.97	0.88	0.61
Renal cell carcinoma	<10	<10	<10	<10	95 (5.4)	0.81	0.79	0.62
Other cancer types								
Colorectal cancer	11 (26.2)	<10	<10	<10	314 (17.8)	0.52	0.38	0.16
Breast	<10	<10	<10	0 (0.0)	177 (10.1)	0.3	0.28	0.26
Prostate	<10	<10	<10	<10	218 (12.4)	0.04	0.15	0.08
Metastatic vs non metastatic disease, N (%)[f]								
Metastatic disease (C77*-C80*)	26 (66.7)	<10	<10	17 (81.0)	1279 (75.1)	0.0001	<0.0001	0.0003
Non-metastatic disease	10 (25.6)	<10	<10	<10	409 (24.0)			

Unknown[g]	<10	<10	<10	<10	16 (0.9)			
Baseline comorbidities (assessed in the 24 months before the index VTE event)								
CCI								
Mean (SD)	6.0 (3.4)	5.3 (2.9)	5.8 (3.2)	6.5 (3.7)	6.7 (3.3)	0.25	0.19	0.17
Median (IQR)	5 (3-9)	4 (2-8)	6 (2-8)	6 (3-10)	8 (3-9)			
CCI 3 or 4, n (%)	<10	0 (0.0)	0 (0.0)	<10	178 (10.1)	0.71	0.53	0.56
CCI >4, n (%)	29 (69.1)	14 (73.7)	<10	15 (65.2)	1267 (71.9)			
History of bleeding (≤24 months before index date), all diagnoses, n (%)	<10	<10	<10	<10	398 (22.6)	0.56	0.78	0.59
History of bleeding (≤24 months before index date), principal diagnosis, n (%)	<10	<10	0 (0.0)	<10	156 (8.9)	0.10	0.72	0.7
Recent history of bleeding (≤3 months before index date), principal diagnosis, n (%)	<10	<10	0 (0.0)	0 (0.0)	92 (5.2)	0.31	0.53	0.41
Comorbidities, n (%)								
Moderate to severe renal disease[h]	<10	<10	<10	<10	113 (6.4)	0.04	0.12	0.04
Pulmonary disease	11 (26.2)	<10	<10	<10	217 (12.3)	0.06	0.03	0.007
Hypertension	19 (45.2)	<10	<10	10 (43.5)	688 (39.1)	0.69	0.70	0.42
Cerebrovascular disease	<10	0 (0.0)	0 (0.0)	<10	105 (6.0)	0.68	0.47	0.75
Diabetes	<10	<10	<10	<10	335 (19.0)		0.61	0.70
Obesity	<10	<10	<10	<10	222 (12.6)	0.10	0.24	0.09
Anemia	14 (33.3)	<10	<10	11 (47.8)	611 (34.7)	0.19	0.09	0.86
Recent history of falls	0 (0.0)	0 (0.0)	0 (0.0)	0 (0.0)	30 (1.7)	0.87	0.70	0.39
Concomitant antiplatelet agent (at index date ± 90 days), n (%)	<10	<10	<10	<10	436 (24.7)	0.77	0.71	0.62
LMWH treatment duration (months)								
Mean (SD)	8.8 (11.1)	8.7 (11.9)	10.6 (13.5)	8.8 (10.7)	9.4 (10.9)	0.71	0.93	0.70
Median (IQR)	3.8 (1.5-11.8)	6.0 (1.2-11.5)	6.7 (1.5-11.5)	3.2 (1.6-14.0)	5.7 (2.1-12.0)			
Time to first bleeding event (months)								
Mean (SD)	8.3 (11.1)	8.3 (11.9)	10.2 (13.5)	8.4 (10.6)	7.6 (10.3)	0.64	0.91	0.66
Median (IQR)	3.3 (0.8-11.2)	5.0 (0.6-11.2)	6.5 (1.2-11.2)	2.9 (1.2-13.6)	3.7 (1.0-9.7)			
Follow-up time (months)[i]								
Mean (SD)	8.8 (11.1)	8.8 (11.9)	10.6 (13.5)	8.8 (10.7)	15.8 (15.7)	0.03	0.02	0.004
Median (IQR)	3.8 (1.5-11.8)	6.0 (1.2-11.5)	6.7 (1.5-11.5)	3.3 (1.6-14.0)	10.1 (4.5-22.2)			

[a] Comparison among patients who switched to a DOAC, switched to a VKA, switched to a parenteral AC, and did not switch: chi square test for categorical variables and ANOVA for continuous variables

[b] Comparison among patients who switched to an oral AC, switched to a parenteral AC, and did not switch: chi square test for categorical variables and ANOVA for continuous variables

[c] Comparison between patients who switched AC and those who did not: chi square test for categorical variables and ANOVA for continuous variables

[d] Based on area of residence at the time of the index VTE event

[e] Gynecologic cancers included malignant neoplasms of the vulva, vagina, cervix uteri, corpus uteri, uterus (part unspecified), ovary, placenta, and other and unspecified female genital organs

[f] Percentages were computed among patients with recorded information on metastasis status. Patients who developed metastatic disease more than 30 days after the index date were excluded from the analysis

[g] ICD-10 information missing
[h] ICD-10: I120, I131, N032-N037, N052-N057, N18, N19, N250, Z490, Z491, Z492, Z940, or Z992; or CCAM procedure codes for dialysis: JVJB001 or JVJB002 [29]
[i] From the index date until censoring or end of follow-up
Percentages are proportions of patients with the given characteristic among patients with the specific AC switch. Data are not shown for patients who switched to a VKA because the number of patients was <10.
Abbreviations: AC, anticoagulant; CCI, Charlson comorbidity index; DOAC, direct oral anticoagulant; DVT, deep vein thrombosis; ICD-10, International Classification of Diseases 10th revision; IQR, interquartile range; LMWH, low-molecular-weight heparin; PE, pulmonary embolism; Q, quintile; SD, standard deviation; VTE, venous thromboembolism
